# Racial disparities in Medicaid home and community-based service utilization and expenditures among persons with multiple sclerosis

**DOI:** 10.1186/s12913-018-3584-x

**Published:** 2018-10-12

**Authors:** Chanee D Fabius, Kali S Thomas, Tingting Zhang, Jessica Ogarek, Theresa I Shireman

**Affiliations:** 1 Center for Gerontology and Healthcare Research, Brown University School of Public Health, Box G-S121(6), 121 S Main Street, Providence, RI 02912 USA; 2 Department of Health Policy and Management, Johns Hopkins Bloomberg School of Public Health, Baltimore, MD USA; 3 Center of Innovation in Long-Term Services and Supports, U.S. Department of Veterans Affairs Medical Center, Providence, RI USA

**Keywords:** Home and community-based services, Disparities, Race

## Abstract

**Background:**

Medicaid home and community-based services (HCBS) provide services such as personal care, nursing, and home-delivered meals to aging adults and individuals with disabilities. HCBS are available to people across racial and ethnic groups, yet racial disparities in Medicaid HCBS utilization and expenditures have been understudied. Individuals with multiple sclerosis (MS) may be particularly impacted by HCBS, as nearly one-third requires assistance at home. The present study examined whether disparities exist in Medicaid HCBS utilization and expenditures among HCBS users with MS.

**Methods:**

We used secondary data to conduct a retrospective cohort analyses including 7550 HCBS recipients with MS. Demographic data was obtained from the Medicaid Analytic eXtract Personal Summary file, Medicaid HCBS service utilization and expenditures were obtained from the Other Therapy file, and comorbidities from the Medicare Chronic Condition Warehouse. Univariate and bivariate statistics were used to describe the sample and provide comparisons of characteristic by race. Logistic regression predicted the likelihood of using HCBS type and gamma regression was used to predict Medicaid HCBS expenditures.

**Results:**

Black HCBS users were younger, more likely to be female, and were more impaired than Whites. Multivariate analyses showed that Blacks were less likely to receive case management, equipment, technology and modification services, and nursing services compared to Whites. Additionally, Black men had the lowest Medicaid HCBS expenditures, while White men had the highest.

**Conclusions:**

Findings shed light on disparities among HCBS users with MS. As Blacks are already disproportionately affected by MS, these results reveal target areas for future research. Future work should examine the factors that contribute to these disparities, as well as determine the extent to which these inequities impact outcomes such as hospitalizations and nursing home admissions.

## Background

In response to an increasing need for community-based care, and under the expansion of the Affordable Care Act (ACA), Medicaid has expanded its support for home and community-based services (HCBS) [1, 2]. HCBS are long-term services and supports (LTSS) that provide cost-saving nursing home alternatives in home and community settings and are administered through states’ Medicaid programs. Services provide care for multiple needs, such as personal care, housekeeping, and transportation [3, 4]. HCBS also provide assistance to people across disabilities, ages, races, and ethnicities, yet racial disparities in services have been understudied, and findings have often produced conflicting results.

One group particularly impacted by the availability of HCBS consists of people with multiple sclerosis (MS), which is a disabling disease of the central nervous system. MS affects 400,000 people across the United States and two million people worldwide [5, 6]. Due the disease’s unpredictable nature, impact on physical and cognitive function, and costly care needs, MS many times results in individuals being enrolled in Medicare before the age of 65, as well as having to spend down their resources to become Medicaid eligible to cover expenses associated with healthcare costs. Persons with MS are also forced to leave the workforce prematurely, further comprising their financial stability [7]. Unfortunately, the confounding of these factors – declining health and increasing functional and financial need – puts people with MS at further risk of nursing home placement [8]. These risks might be mitigated by the use of HCBS, which have been found to reduce the use of long-stay nursing home care [9].

Findings suggest racial differences in MS: on average, Blacks are diagnosed at a younger age, and their disease course is more rapid compared to Whites [6, 10]. Blacks with MS are also more likely to be women, and to have higher MS severity scores compared to Whites after controlling for age, gender, and insurance type [11]. Additionally, a smaller proportion of Blacks are treated or evaluated at MS centers, clinics, or neurologists compared to both White and Latinos [12]. These differences may make Blacks with MS more susceptible to needing assistance from HCBS. Some findings point to Blacks more often using HCBS than Whites to avoid nursing home placement [13, 14]. Other research supports the opposite, with Blacks using services less than Whites [15]. Studies have traditionally been limited to smaller samples with self-reported data, making it difficult to generalize findings to larger populations. Additionally, most studies have limited findings to reporting the overall utilization of HCBS. Research is needed to understand whether disparities exist in the types of services provided to HCBS users. Findings might reveal racial differences in preferences for care, as well as issues with access to specific services.

Disparities in HCBS utilization and Medicaid spending may have larger implications for long-term care, as rates of nursing home admission vary among race groups [2] and may be a result of a combination of factors. For instance, inadequate access to specialty care, mistrust of medical professionals, and cultural and religious beliefs may impact the decision to use health services [6, 16]. Further, prior studies have shown that Medicaid HCBS spending reduces institutional spending [17, 18]. However, studies have also shown that Blacks have lower overall Medicaid expenditures as well as HCBS spending compared to Whites among groups with different healthcare needs [19, 20]. For the MS population, disparities in utilization and Medicaid spending may contribute to adverse outcomes, such as nursing home admissions and other health service utilization (e.g. hospitalizations).

With the lack of research on disparities in both MS and HCBS, as well with what is known about differential rates of nursing home admission among Black and White adults, a necessary step in research is to understand differences in patterns of use of HCBS. We ask specifically, among HCBS users, are there racial disparities in utilization and expenditures? We hypothesize that there will be significant disparities in HCBS utilization and expenditures, although we hold no assumptions about the direction of the relationships between race, services and expenditures.

## Methods

We used secondary data to conduct a retrospective cohort analysis including dual-eligibles with MS for years 2010–2012. We obtained demographic data from the Medicaid Analytic eXtract Personal Summary (MAX PS) file, Medicaid HCBS service utilization and expenditures from the Other Therapy (MAX OT) files, and comorbidities from the Medicare Chronic Condition Warehouse (CCW).

### Sample

The study participants included 99,064 Medicaid-eligible individuals with MS. Individuals were excluded if they were not Medicare and Medicaid dually-eligible for at least 6 months (40,860), had a long-term stay in a nursing facility or were in a nursing facility at the start of the 7th month of dual eligibility (2546), or were categorized as another race/ethnicity (American Indian, Asian-American, Pacific Islander, Hispanic) (6276). These exclusion criteria left a sample of 49,382 (non-Hispanic) White and (non-Hispanic) Black dual-eligibles. We further reduced the sample to those who received HCBS, based upon a published HCBS taxonomy [21] and the MAX OT file. We, therefore, categorized HCBS utilization and Medicaid expenditures for a final analytic sample of 7550 people.

### Study outcomes

#### HCBS categories and expenditures

We used the MAX OT file to organize HCBS procedure codes for people with Medicaid program types 6 (home and community-based care for disabled elderly and individuals 65 and older) and seven (home and community-based care waiver services) into HCBS taxonomy categories [21]. The taxonomy consists of 18 categories (capturing over 60 specific services) and was created to address the ongoing challenge of studying HCBS expenditures and utilization. For our sample, we were able to categorize services into the following 13 taxonomy categories: case management, round-the-clock services, supported employment, day services, nursing, home-delivered meals, home-based services, caregiver support, other health and therapeutic services, participant training, equipment, modifications, and technology, and non-emergency transportation services. Each taxonomy category was treated as a dichotomous measure. To calculate Medicaid HCBS expenditures, we created an aggregate file that summed Medicaid expenditures across HCBS taxonomy categories for each beneficiary. This process yielded a monetary value for each taxonomy category, as well as total Medicaid expenditures, per beneficiary.

### Independent variables

Race, our variable of interest, was dichotomous (White or Black). Age was included as a continuous variable. We included several comorbidities that are common in people with MS and have significant effects on MS disability progression and health services as dichotomous variables: anxiety, epilepsy, heart failure, COPD, depression, hypertension, hyperlipidemia, heart disease, and mobility impairment [22, 23]. We created a multiple category variable to represent state of residence, as well as a variable representing the natural logarithm of months of eligibility per beneficiary (6 months minimum).

### Statistical analysis

We used univariate statistics to describe our total sample, and chi-square tests and independent samples t-tests to determine significant racial differences in HCBS utilization and expenditures among HCBS users by race. Next, we assessed the odds of utilization for the five services with the most utilization (case management; equipment, technology, and modifications; home-based care; home-delivered meals; nursing) using logistic regression, controlling for demographic characteristics, comorbidities, state, and months of eligibility. We initially attempted logarithmic transformations on expenditures to use linear regression to predict total Medicaid HCBS expenditures, because the test for normality (Kolmogorov-Smirnov) indicated that the distribution of expenditures was skewed (*p* = .000). The data remained skewed - as a result, we used a gamma generalized linear model (GLM) with a log-link function. Gamma regression has been tested and recommended in the analyses of skewed healthcare cost data [24]. People with the highest expenditures (greater than $447,830) were removed from our multivariate analyses to eliminate further skewing of the data (*n* = 26). All regression models included an interaction term between race and sex to determine whether the effects of race on HCBS utilization and expenditures varied by sex. To account for variation between states, we adjusted for standard errors by clustering beneficiaries within states using Stata’s cluster subcommand. To adjust for time, we included the log of months of Medicaid eligibility per beneficiary as an offset variable based on the assumption that the likelihood of service utilization would not change over time. For all statistical tests, significance was determined at the α = .05 level. All analyses were completed using Stata 15.0.

## Results

Table 1 provides the descriptive characteristics for the total sample (*N* = 7550) as well as according to race. The average age of the 7550 HCBS recipients was 54 (SD = 12) years. Approximately three-quarters were female. Depression was the most prevalent comorbidity, reported by 66% of the sample. Other comorbidities with a high prevalence included: hypertension (63%), hyperlipidemia (50%), and mobility impairment (43%). Differences by race were observed for all characteristics with the exception of epilepsy. Black HCBS users were younger, more often female, and had higher rates of heart failure, diabetes, hypertension, and mobility impairment compared to Whites. White HCBS users were more likely to have anxiety, COPD, depression, and hyperlipidemia.

**Table 1:** Characteristics of MS Patients who Receive Medicaid Home and Community-Based Services, by Race

Baseline characteristics	Total 100% (7550)	White 75% (5657)	Black 25% (1893)	*p*-value
Age, M (SD)^a^	54 (12)	55 (12)	52 (14)	.000
Gender, %^b^				
Female	74	73	76	.005
Male	26	27	24	
Months of eligibility, M (SD)^a^	9 (5)	9 (5)	9 (6)	.262
Selected chronic conditions, %^b^				
Anxiety	33	36	24	.000
Epilepsy	15	15	15	.966
Heart failure	26	25	30	.000
Chronic obstructive pulmonary disorder	31	33	24	.000
Depression	66	70	56	.000
Diabetes	34	32	38	.000
Hypertension	63	61	70	.000
Hyperlipidemia	50	51	46	.000
Heart disease	34	34	36	.038
Mobility impairment	43	41	49	.000
Total # of chronic conditions, M (SD)^a^	4 (2)	4 (2)	4 (2)	.176

*Note: Abbreviation*: *SD* standard deviation

^a^Student’s t-test

^b^Chi-square test

**Fig. 1:**
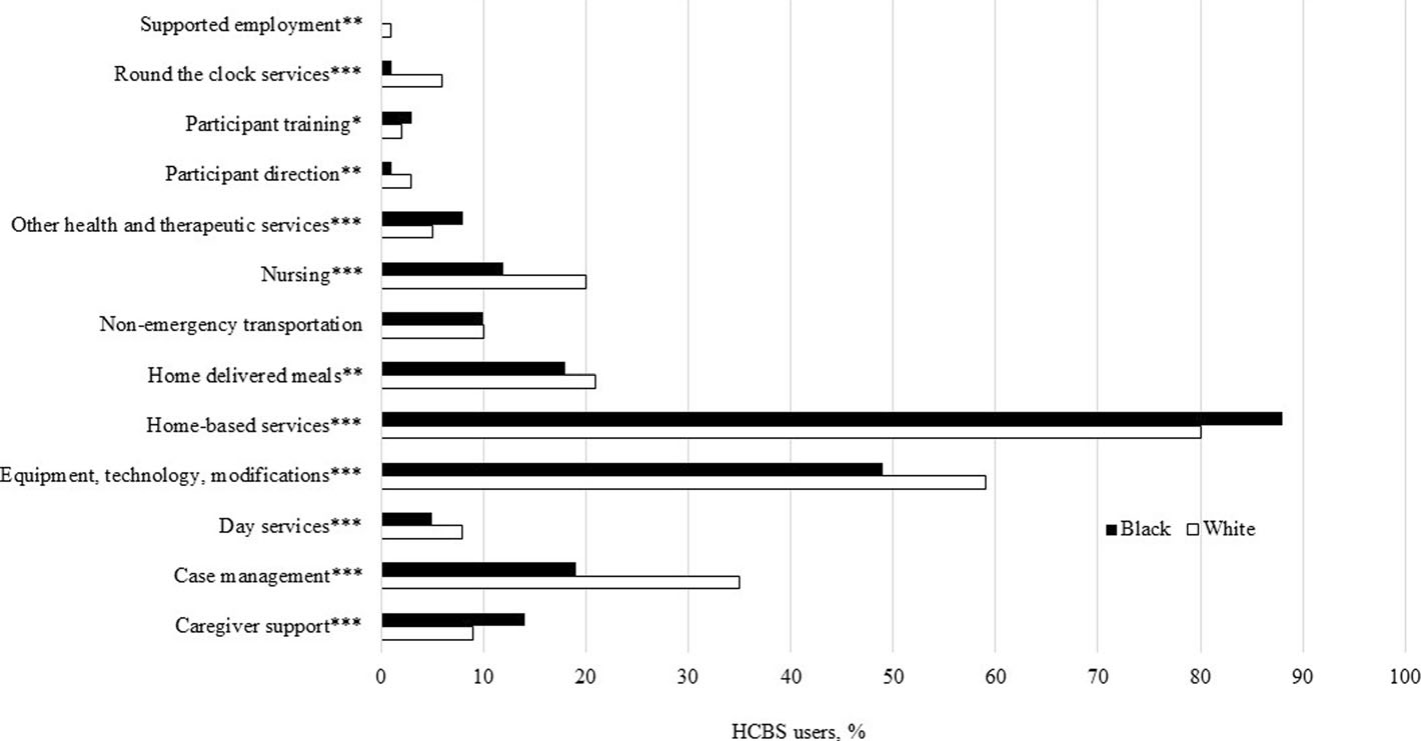
Comparison of Type of HCBS Utilization among HCBS Users, by Race. *Note*: *p < .05; **p < .01; ***p < .001

**Fig. 2:**
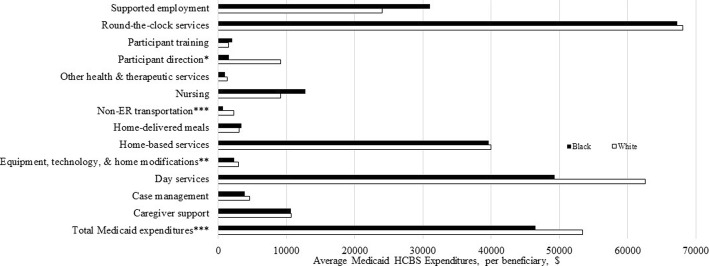
Average Medicaid HCBS Expenditures per User, by Race. *Note*: *p < .05; **p < .01; ***p < .001

Racial differences in HCBS type among HCBS users were observed for all HCBS categories with the exception of non-emergency transportation (Fig. 1). Whites were more likely than Black to use all services, with the exception of home-based services, caregiver support, and other health and therapeutic services. We also compared average total Medicaid HCBS expenditures per user by race (Fig. 2). Differences by race were observed for equipment, technology, and modifications, non-emergency transportation, participant direction, and total expenditures. Whites had higher expenditures compared to African-Americans across these categories.

**Fig. 3:**
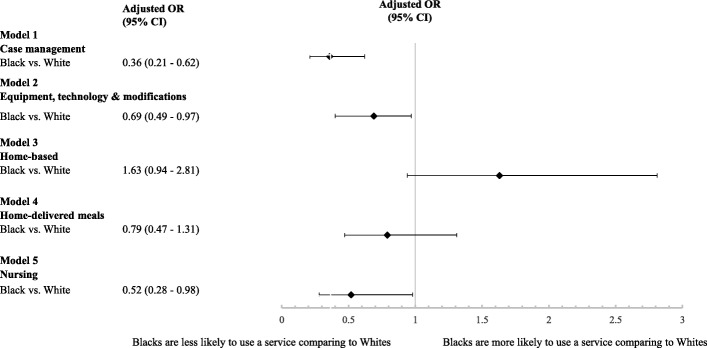
Racial Disparities in HCBS Utilization. *Note*. Logistic regression was used to assess the relationship between race and services for each of the five models. Each model adjusted for age, sex, anxiety, epilepsy, heart failure, COPD, depression, diabetes, hypertension, hyperlipidemia, ischemic heart disease, mobility impairment, race by gender interaction, state, and months of eligibility *p < .05; **p < .001

Figure 3 presents the results of our logistic regression models evaluating the association between race and the likelihood of using case management, equipment, technology, and modification, home-based, home-delivered meals, and nursing services among HCBS users. After controlling for age, sex, comorbidities, state, and months of eligibility, Black were 64% less likely than Whites to used case management, 31% less likely to use equipment, technology, and modification services, and 48% less likely to use nursing services. The relationship between race and home-based services and home-delivered meals was no longer significant. The race by sex interaction term was not significant for any of the models.

**Fig. 4:**
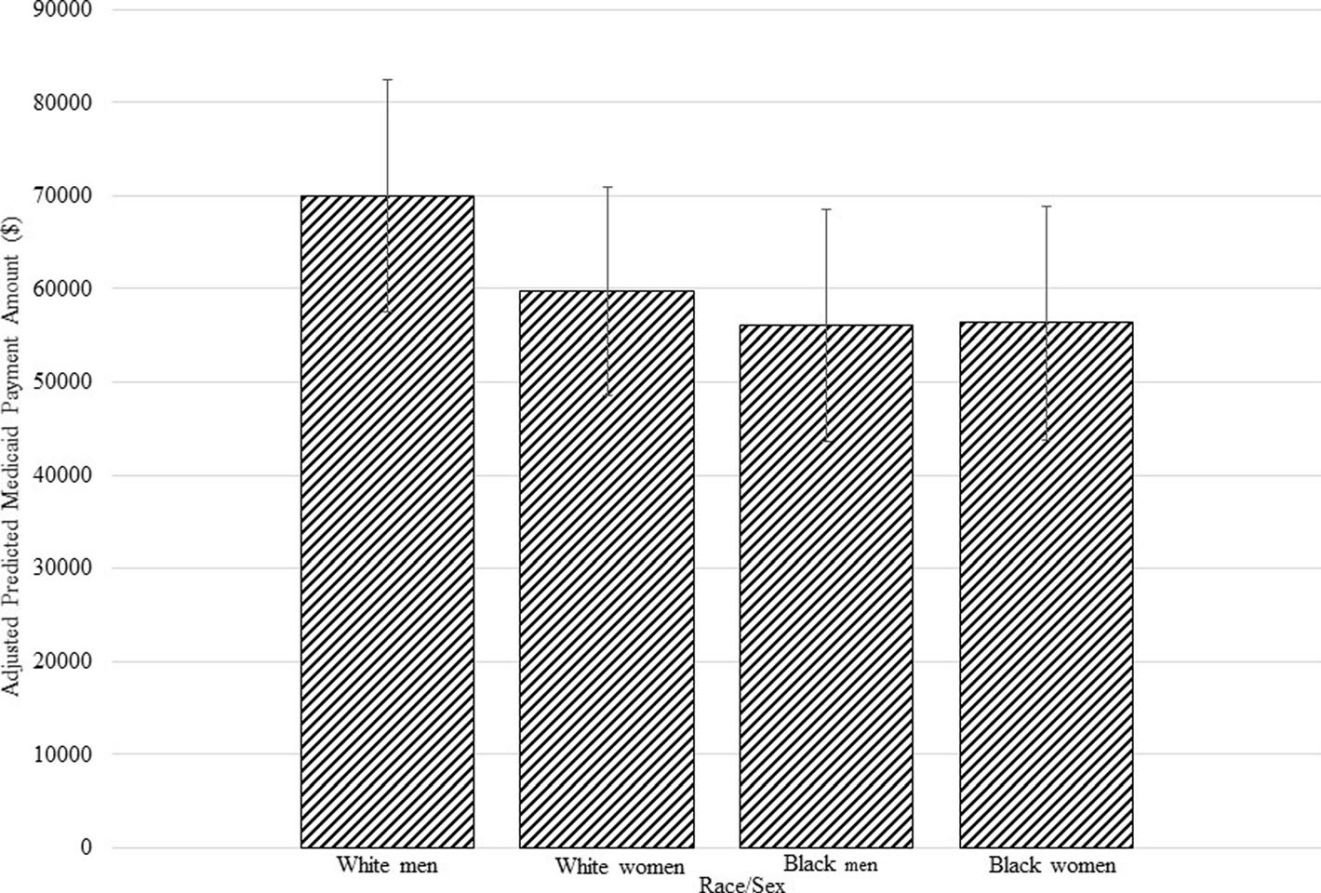
Medicaid HCBS Expenditures, by Race and Sex . *Note.* Adjusted probability of total Medicaid HCBS expenditures (*p* < .001). Model adjusted for age, anxiety, epilepsy, heart failure, COPD, depression, diabetes, hypertension, hyperlipidemia, ischemic heart disease, mobility impairment, state, and months of eligibility

As to the association between race and total expenditures, we first present findings from our multivariate regression predicting Medicaid HCBS expenditures. Table 2 lists the parameter estimates for the gamma GLM regression. Coefficients are representative of the change in the log of expenditures expected with an increase of one unit of an independent variable and are interpreted as the multiplicative effects on the dependent variable. As an example, women are predicted to have Medicaid HCBS expenditures that are exp. (−.17), or roughly 16% lower than that of men. Additionally, an individual with mobility impairment is predicted to have expenditures that are exp. (.29), or 34% higher than someone without mobility impairment. Other predictors of decreased expenditures include increased age, Black race, and having a diagnosis of anxiety or heart disease were all associated with lower expenditures. In addition to mobility impairment, epilepsy was the only other covariate associated with higher expenditures. Our results also indicate a positive and significant relationship between a race by sex interaction and Medicaid HCBS expenditures, presented in Fig. 4, where the adjusted predicted values of total Medicaid HCBS expenditures by race and sex and 95% confidence intervals are shown. After controlling for covariates, Black men had the lowest predicted HCBS expenditures ($56,088.17), followed by Black women, ($56,335.60), and White women ($59,783.15). White men had the highest total Medicaid HCBS expenditures ($70,002.33).

**Table 2:** Determinants of Medicaid HCBS Expenditures^a^

Characteristics	Coefficient	CI	*p*-value
Age	−.01	[−.01, −.00]	.011
Female^b^	−.16	[−.27, −.04]	.008
Black^c^	−.22	[−.40, −.05]	.014
Selected chronic conditions			
Anxiety	−.08	[−.12, −.03]	.002
Epilepsy	.34	[.15, .53]	.000
Heart failure	.00	[−.06, .01]	.997
Chronic obstructive pulmonary disorder	.02	[−.04, .08]	.478
Depression	.06	[−.03, .14]	.212
Diabetes	−.01	[−.09, .07]	.857
Hypertension	−.09	[−.20, .02]	.096
Hyperlipidemia	.01	[−.05, .07]	.707
Heart disease	−.09	[−.15, −.02]	.009
Mobility impairment	.31	[.19, .43]	.000
Race x Sex	.16	[.00, .32]	.045
Intercept	9.17	[8.84, 9.49]	.000

Note

^a^GLM with Gamma distribution and log link function

^b^Reference group for female is male

^c^Reference group for Black is White

## Discussion

Our findings support our hypotheses that there are disparities in HCBS utilization as well as Medicaid expenditures among MS patients who receive HCBS. Specifically, we found that among HCBS users, for the five services with the most utilization, after controlling for demographic characteristics, chronic conditions, state, and months of eligibility, Whites were more likely to use case management, equipment, technology, and modifications, and nursing services. We also found significant differences in the association between race and Medicaid HCBS expenditures when we stratified our analyses by sex. White men had the highest HCBS expenditures, followed by White women and African-American women. Black men had the lowest Medicaid HCBS expenditures.

Case management services are a method of matching clients to appropriate services through assessment, care plan development, coordination, and arrangement of HCBS [25]. Our results align with prior findings on racial differences in case management preference. In a study examining how preferences for consumer direction vary by race and ethnicity, Black who desired more control over home care workers were also less likely to prefer a traditional model of case management, in which an agency takes responsibility to develop an individual’s service plan and choosing and arranging home care services. Rather, they were more likely to prefer either a cash and counseling or negotiated care management model. Both of these models of care give HCBS recipients more freedom to choose services by providing a monthly budget and some assistance to arrange home care services [26]. While this may be the case, most states do not allow users the option of refusing case management services because they are an integral part of the HCBS waiver [27]. Further research should be done to understand the disparity in utilization.

The equipment, technology, and modification taxonomy category covers a range of services, such as home and vehicle modifications, as well as personal products (e.g. incontinence products). However, for those in our sample who used services in this category, about 90% of procedure codes categorized as equipment, technology, or modification services were for home modifications. It isn’t entirely clear why Blacks are less likely to receive home modifications, but we hypothesize two scenarios that may offer explanations. First, Blacks may already be living in apartments, which are more likely to already have ramps and wheelchair access available and may not need additional HCBS resources to modify their homes [28]. Conversely, Blacks are less likely to be homeowners [29], so they may be unable to modify homes due to restrictions enforced by landlords. While provisions such as the Fair Housing Act protect against discrimination based on race and disability for housing-related transactions, it is likely difficult to modify an apartment after someone is already living there [30]. For people with a condition with a worsening disability trajectory, landlords have to consider that individuals requiring additional home modifications may eventually leave their independent living arrangement to live with family or be admitted to a nursing home.

Blacks were also less likely to use nursing services, even after adjusting for demographic characteristics, comorbidities, state, and length of eligibility. This finding appears to follow a pattern – one study showed that among older adults with diabetes received home health services, Blacks received fewer skilled nursing visits compared to White [31]. This might be due to a few different reasons that are explained by challenges in the provider-patient relationship, such as provider bias or recipient distrust. Doctors and nurses have acknowledged that provider bias contributes to disparities in care [32]. However, bias is also influenced by challenges with patients – Blacks are more likely to not adhere to medical instructions [33]. The combination of these factors likely contributes to the lower likelihood of Blacks receiving nursing services.

While we find that several factors contribute to Medicaid HCBS expenditures, our findings indicate racial and gender disparities. Black men had the lowest Medicaid HCBS expenditures. While Black men made up only 6% of our sample, were younger, and had fewer comorbidities compared to White men and women, and Black women, they had the highest rates of mobility impairment, which is typically attributed to a greater need for HCBS. This finding is important, as Black men incur greater healthcare utilization costs (e.g. inpatient, emergency room, outpatient, prescription drugs) than any other racial/ethnic group. [34]. These costs might be reduced if HCBS utilization increased for Black men.

### Limitations

While our findings provide evidence for racial differences in HCBS utilization and Medicaid HCBS expenditures among dually-eligible persons with MS, we acknowledge several limitations. First, our findings reflect utilization and expenditures for a small sample of dual-eligible adults in the MS population, which could present challenges with generalizability. However, our sample resides in 48 states and Washington DC, therefore having implications for national Medicaid policy implementation. Second, this study presents findings from the MS population; results might be different for other chronic conditions. Still, the needs of the MS population may reflect that of aging adults in need of HCBS, although our sample members were younger than typical HCBS recipients. Third, as our data come from Medicare and Medicaid claims, we could not include variables, such as socioeconomic status or other social-environmental factors that might better explain some of our findings. For example, HCBS utilization is heavily influenced by the presence (or lack thereof) of an informal care network. Future studies should include characteristics that reflect systems of social support as well as the social environmental context in which people are living. Fourth, for our multivariate analyses, we assumed that the likelihood of service utilization would not change over time. We are unable to determine whether this is true in all cases, and HCBS utilization may be impacted by a number of unobserved variables (e.g. availability of informal care, MS disease course and disability progression). Last, we limited our study to White and Black individuals with MS – Hispanics represented roughly 5 % of our sample, as well as individuals identifying as other races/ethnicities, including American Indian, Asian-American and Pacific Islander. While incidence of MS is lower in other racial/ethnic groups compared to Whites and Blacks, differences may exist in HCBS utilization, as some research has shown that MS treatment choices vary across racial and ethnic groups [6].

## Conclusion

Findings from this study suggest that there are racial disparities in case management, equipment, technology, and modification services, nursing services, and Medicaid HCBS expenditures for Black and White MS dual-eligibles. As African-Americans are already disproportionately impacted by MS, findings from this study reveal areas that warrant additional examination. Future research should make an effort to better understand the behavioral, cultural, and social mechanisms that may contribute to disparities in utilization. Next steps should include determining the effect of disparities in Medicaid HCBS spending on nursing home admissions and hospitalizations among persons with MS. Medicaid HCBS support persons with MS in the community. With roughly one-third of the MS population requiring assistance at home, there will be ongoing opportunities to interrupt and correct the disparities that are presently responsible for the gap in the disease trajectory and management of MS.
